# Transforming educator expectations in public health, nursing, and medicine: a two-phase survey on digital tools in education

**DOI:** 10.1186/s12909-025-08406-x

**Published:** 2025-12-08

**Authors:** Tom K. Schaal, Maria A. Marchwacka, Daniel Tolks, Joachim Kugler

**Affiliations:** 1 Faculty of Health and Healthcare Sciences, University of Applied Sciences Zwickau, Zwickau, Germany; 2 Faculty of Healthcare, Ostfalia University of Applied Sciences, Wolfsburg, Germany; 3 International University Hamburg, Hamburg, Germany; 4 Medical Faculty Rostock, Rostock, Germany; 5 Department of Public Health, Dresden Medical School, University of Dresden, Dresden, Germany

**Keywords:** Digital transformation, Digital teaching, Higher education

## Abstract

The digital transformation of healthcare education has accelerated significantly due to the COVID-19 pandemic, shifting traditional, practice-oriented teaching towards digital methodologies. Before the pandemic, digital teaching was minimally adopted and lacked systematic strategies, especially in Germany’s higher education institutions. This study comprised two cross-sectional online surveys among university faculty in public health, nursing, and medicine—conducted in 2020 and 2021—with a total of 238 participants. The surveys explored the use of digital tools, satisfaction with digital teaching, perceived challenges, and expectations for future teaching practices. Digital platforms such as Zoom, Moodle and MS Teams emerged as the most widely used tools, primarily for content delivery rather than interactive or collaborative activities. Faculty reported generally positive experiences and increasing adaptation to digital teaching, though concerns persisted regarding its time demands and limited support for personalized learning and student engagement. Satisfaction with digital teaching grew between the two survey phases, yet remained moderate regarding didactic support and student outcomes. Educational videos and learning management systems were highlighted as favored methodologies. Nonetheless, reservations about reducing attrition rates and supporting disadvantaged learners indicate unresolved challenges. While digital methodologies in healthcare education have gained acceptance, their integration remains shaped by technical considerations rather than didactic innovation. To optimize digital teaching, institutions must provide targeted professional development, define clear frameworks for digital competencies, and implement systematic evaluation processes. The adoption of emerging technologies, including artificial intelligence, necessitates further investment in faculty training and attention to ethical and legal considerations. Comprehensive pedagogical reforms are essential for the sustainable advancement of digital education in healthcare.

**Supplementary Information:**

The online version contains supplementary material available at 10.1186/s12909-025-08406-x.

## Introduction

The digital transformation of higher education in healthcare has undergone a marked acceleration, predominantly driven by the emergency response necessitated by the COVID-19 pandemic [1, 2]. Conventionally, healthcare education has relied predominantly on face-to-face, practice-oriented teaching methodologies, emphasizing clinical skills, patient interaction, and experiential learning [3–7].

However, prior to the advent of the pandemic, the adoption of digital methodologies in pedagogical practice remained in its infancy. As demonstrated in the German research by Winde et al. [8] and Gilch et al. [9], the number of courses offered digitally prior to the advent of the pandemic was minimal, with a mere 12% of courses being delivered digitally. Furthermore, only 14% of German higher education institutions had a defined digitization strategy in place [8, 9]. The sudden shift to emergency remote teaching has necessitated a rapid adaptation of pedagogical approaches by universities and faculties. This transition has brought to light both significant potential and existing gaps in these approaches. This transition presented significant challenges, particularly in light of the deeply entrenched culture of physical presence in healthcare education. The pressing nature of the situation precipitated the swift implementation of a variety of digital teaching methodologies. The specific approaches adopted were contingent on a number of factors, including the availability of resources, the digital competencies of faculty members, infrastructure, and the constrained timeframe [10, 11].

Whilst a full evaluation of these digital semesters is still pending, preliminary assessments have indicated that lecturers, universities and students have expressed a general level of satisfaction with the developments. It is noteworthy that 75% of lecturers reported positive experiences with digital teaching, despite 49% of these having conducted digital teaching for the first time [8]. A recent systematic review on online learning presents a heterogeneous picture: A survey of relevant studies revealed that 36% concluded online learning to be effective, 52% indicated negative outcomes, and 12% reported neutral findings [12]. It is important to note that studies which employ higher methodological standards (e.g. randomized controlled trials) have consistently demonstrated positive learning outcomes. Conversely, studies which assess student satisfaction have predominantly reported negative perceptions.

In Germany, various pilot projects have successfully investigated the integration of digital teaching methods into person-centered curricula, with approaches such as communication training, simulation-based patient interactions, and online examinations [13, 14]. However, findings from recent studies reveal that the digital transformation in academic institutions has so far only been partially achieved. This limited progress is attributable to several obstacles, primarily inadequate communication, insufficient coordination, and ambiguous roles and responsibilities. As a result, there is a clear and growing need for more precisely defined concepts as well as practical guidelines to support the development and implementation of effective digital strategies. These insights underscore the pressing need for comprehensive pedagogical reforms in healthcare higher education, with a particular emphasis on the systematic integration of digital competencies and innovative teaching methodologies. The present study thus examines faculty members’ perceptions and evaluations of the shift towards digital teaching in healthcare, nursing and medicine, identifies preferred digital tools, and derives critical implications for future pedagogical strategies in healthcare education.

## Methods

Two independent, advertising-free, cross-sectional surveys were conducted via www.soscisurvey.de for scientific purposes. The first survey occurred from June to August 2020 and the second from June to July 2021. The online survey was designed by the Teaching Section of the German Public Health Association (DGPH) and the Digitization Committee of the Society for Medical Education (GMA), with participation from the Education and Counseling Section of the German Nursing Association (DGP) and the Teaching Working Group of the German Society for Medical Sociology (DGMS). This collaboration allowed for the inclusion of university and college faculty from the disciplines of public health/health sciences, health education, medicine and nursing [15].

This study was conducted exploratively in order to gain new insights and systematically investigate the research area. Due to a lack of comparable data, it was not possible to determine the sensitivity of potentially one-sided hypotheses, which would have been necessary for estimating the sample size and controlling the beta error. Since the analysis is purely descriptive, there are no inferential statistical evaluations of hypotheses or in-depth discussions of error types. Therefore, the focus was primarily on generating trends and identifying key questions to serve as a guide for future research. The questionnaire allowed questions to remain unanswered to avoid a high dropout rate from forced responses. Missing values were not replaced. Approximately 1,700 members of the aforementioned organizations received access to the survey via a web link within their respective society’s newsletter. Personalized participation links were not utilized due to data protection regulations regarding member data. Participants were informed of the objectives, voluntary participation, and anonymous data processing, and were required to give their consent to data protection before proceeding. A comprehensive data protection concept was available for download. The survey adhered to the guidelines for ensuring good scientific practice [16]. The convenience sample of the initial survey comprised 100 university teachers (i.e. professors, research assistants and lecturers). The second survey included 138 university teachers. The participants in the two surveys could not be linked. The selection of a convenience sample for the online survey was based on pragmatic and methodological considerations. By distributing the survey through the newsletters of the four designated umbrella associations, it was possible to reach a broad spectrum of the target population across different professional backgrounds and regions. In the context of Germany, a direct recruitment according to professional qualifications was not feasible, as there are no central registries available for such purposes. Consequently, leveraging the associations provided the most efficient and inclusive means of engaging potential participants, ensuring that findings reflect the diversity present within the field. The data were prepared and analyzed using IBM Statistics SPSS for Mac (version 27).

The survey’s objective was to describe and assess digital teaching from the perspective of faculty who redesigned face-to-face teaching due to the COVID-19 pandemic in the first and third digital semesters, and to descriptively identify change trends. The employed questionnaire was based on a standardized survey predominantly featuring closed-ended questions, systematically collecting extensive data on various aspects of digital teaching. Core themes included the use and application of digital technologies in education, digital teaching and learning concepts, tools for digital instruction, as well as the experiences, attitudes, and challenges faced by instructors regarding digital teaching methods. Additionally, evaluation concepts and data protection issues were addressed. The design of the questionnaire was guided by the “Monitor Digitale Bildung” of the Bertelsmann Foundation from 2017, which first provided a comprehensive and representative empirical data basis on the state of digitized learning in different educational sectors in Germany. The aim is to address key questions regarding the promotion of digital learning processes, the support of disadvantaged learners, and the sustainable qualification of educators. Thus, the survey focuses on the potential of digital technologies in education, their impact on didactic concepts, and the improvement of access to educational opportunities [17, 18]. The validity of the Bertelsmann Gesundheitsmonitor is ensured through a transparent methodology, including the public availability of questionnaires, methodological reports, and raw data, allowing external researchers to verify and independently process the results. Survey findings undergo critical validation by independent experts, and the study’s authors are predominantly affiliated with academic institutions, confirming their independence from the Bertelsmann Foundation. Comprehensive disclosure and accessibility of results enable scientific scrutiny and discussion, collectively strengthening the credibility and external validity of the Bertelsmann Gesundheitsmonitor [17]. Both online surveys were functionally and linguistically checked and adapted in a pretest. The second online survey was supplemented with content relating to future digital orientation in the “New Normal-Teaching”.

## Results

In the first survey, 100 and in the second survey 138 teachers from the health sector took part. The questionnaire was answered by 60 women, 38 men and 2 diverse persons in the first survey and by 88 women and 50 men in the second survey. Those working in education demonstrated a diverse range of characteristics within their professional groups. In both surveys, research assistants constituted the largest group of participants (47 in the first survey, 69 in the second), followed by professors (32 and 38, respectively). The lowest participation rates were observed among lecturers (13 in the first survey, 19 in the second) and teachers with special responsibilities (5 in the first survey, 1 in the second). Other professional groups accounted for 3 votes in the first survey and 11 votes in the second. These results highlight a considerable dominance of medical professionals in the respondent pool, while professionals from health education and teacher education remain strongly underrepresented. For future research, a more balanced participation across all disciplines is recommended to capture a broader range of perspectives. Participants were asked to specify the academic disciplines in which they teach, with multiple responses permitted. In both surveys, medicine was indicated most frequently, followed by public health/health sciences. In contrast, health education was the least commonly reported discipline in the first survey, while teacher education was least frequently selected in the second survey (Table 1).

**Table 1:** Teaching discipline (multiple answers possible)

Courses	Frequency (absolute)	
	* 1st survey*	*2nd survey*
Public Health/Health Sciences	28	48
Health Education	10	12
Medicine	53	82
Nursing	24	26
Teacher Education	14	11

Participants with less than one year of professional experience were the least represented in both surveys. In contrast, the group with 11–20 years of professional experience was the most frequently represented (Table 2).

**Table 2:** Experience as a lecturer/teacher in years

Experience	Frequency (absolute)	
	*1st survey (n* = *100)*	*2nd survey (n* = *138)*
less than one year	8	7
1–5 years	20	22
6–10 years	17	28
11–20 years	29	43
21 years and longer	26	38

**Fig. 1:**
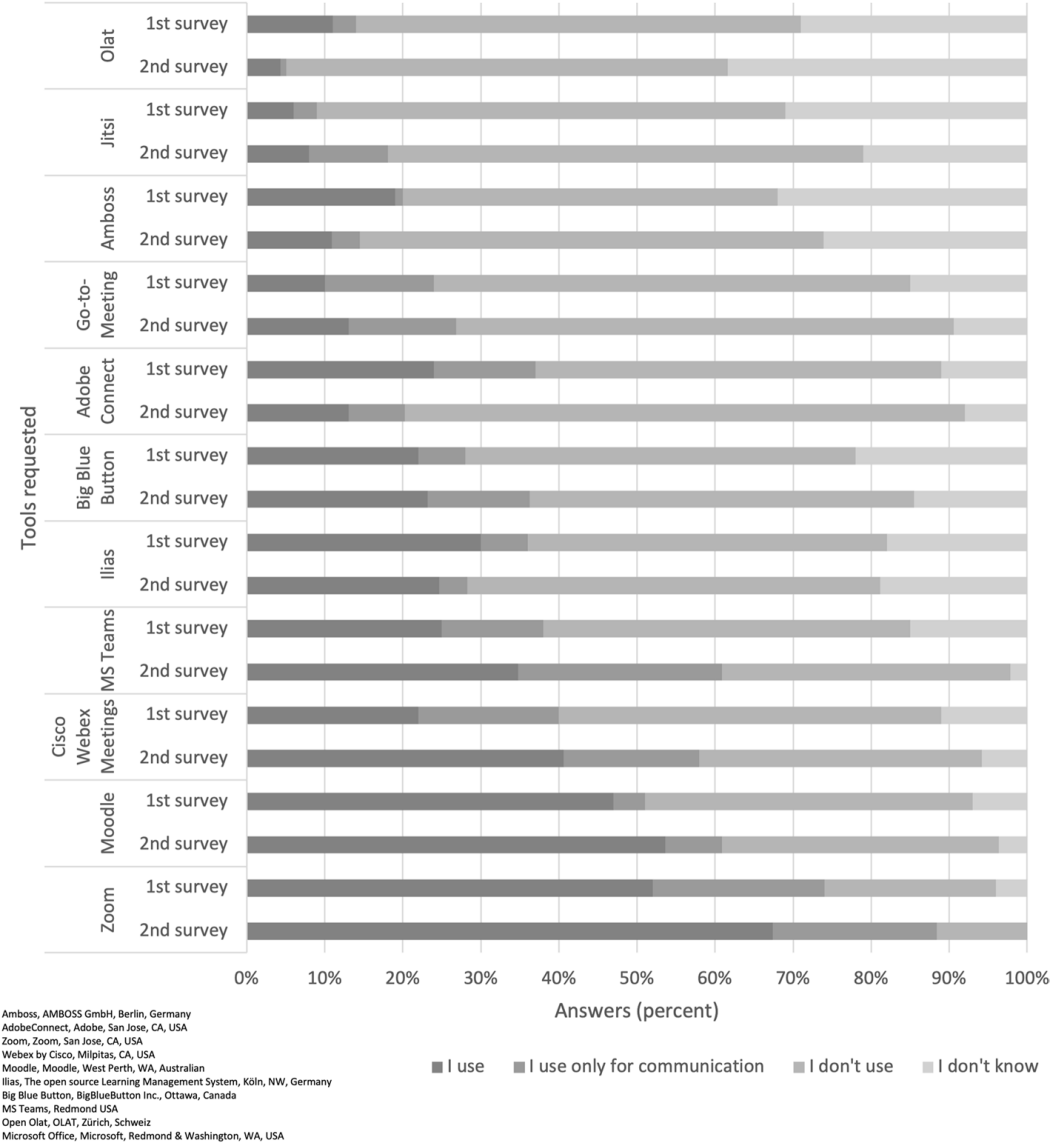
Tools for digital teaching

The most commonly used digital platforms in the analysis (Fig. 1) were Zoom, Moodle and MS Teams.

**Fig. 2:**
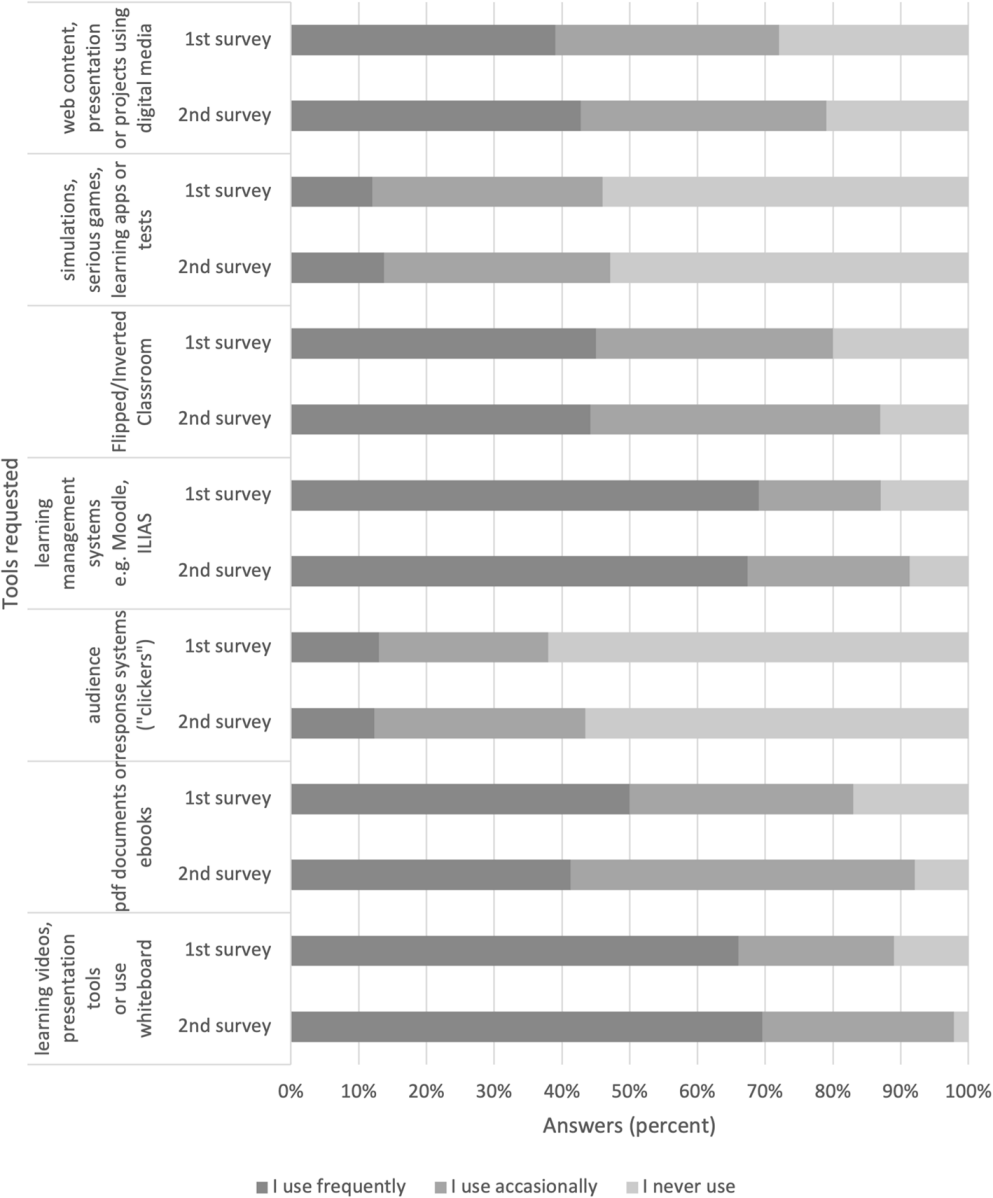
Use of digital offerings

The utilization of digital media by faculty members was predominantly focused on content dissemination rather than on interactive digital or collaborative educational activities, such as audience response systems, simulations, learning apps (Fig. 2).

**Fig. 3:**
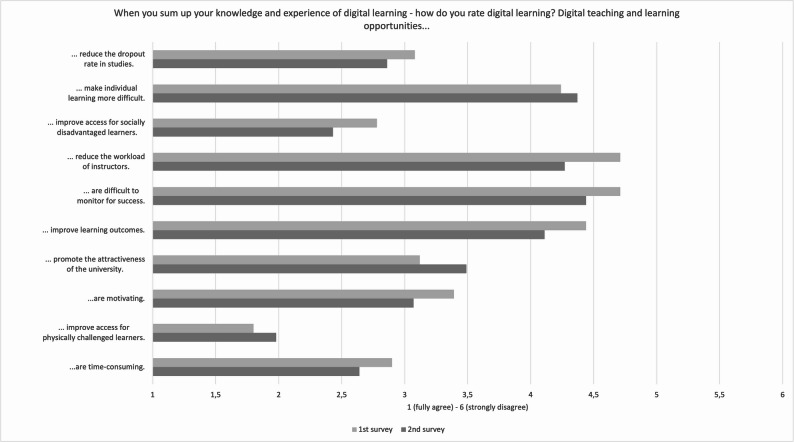
Evaluation of digital teaching (mean value)

A six-point scale was utilized to ascertain how university teachers encapsulate their understanding and practical experience concerning the utilization of digital technologies in educational contexts (Fig. 3). The outcomes of this investigation suggest a notable consensus among the surveyed participants that the employment of digital teaching methodologies is often characterized by a substantial investment of time. Furthermore, positive perceptions were noted for improving accessibility for physically challenged learners, enhancing motivation, and increasing institutional attractiveness. In contrast, the assertion that digital teaching and learning provisions contribute to a reduction in the rate of attrition received the least consensus. Respondents expressed comparatively lower levels of agreement with regard to the capacity of digital education to enhance learning outcomes or to effectively monitor student success. Moreover, concerns were raised regarding the capacity of digital formats to alleviate the workload of instructors or to enhance access for students from disadvantaged socio-economic backgrounds. The divergence of opinion was evident in responses to statements proposing that digital teaching methods impede individual learning and contribute to elevated rates of attrition within academic programs. These results underscore the faculty’s nuanced perspectives, demonstrating a simultaneous recognition of digital education’s benefits and awareness of its persistent limitations and challenges.

**Fig. 4:**
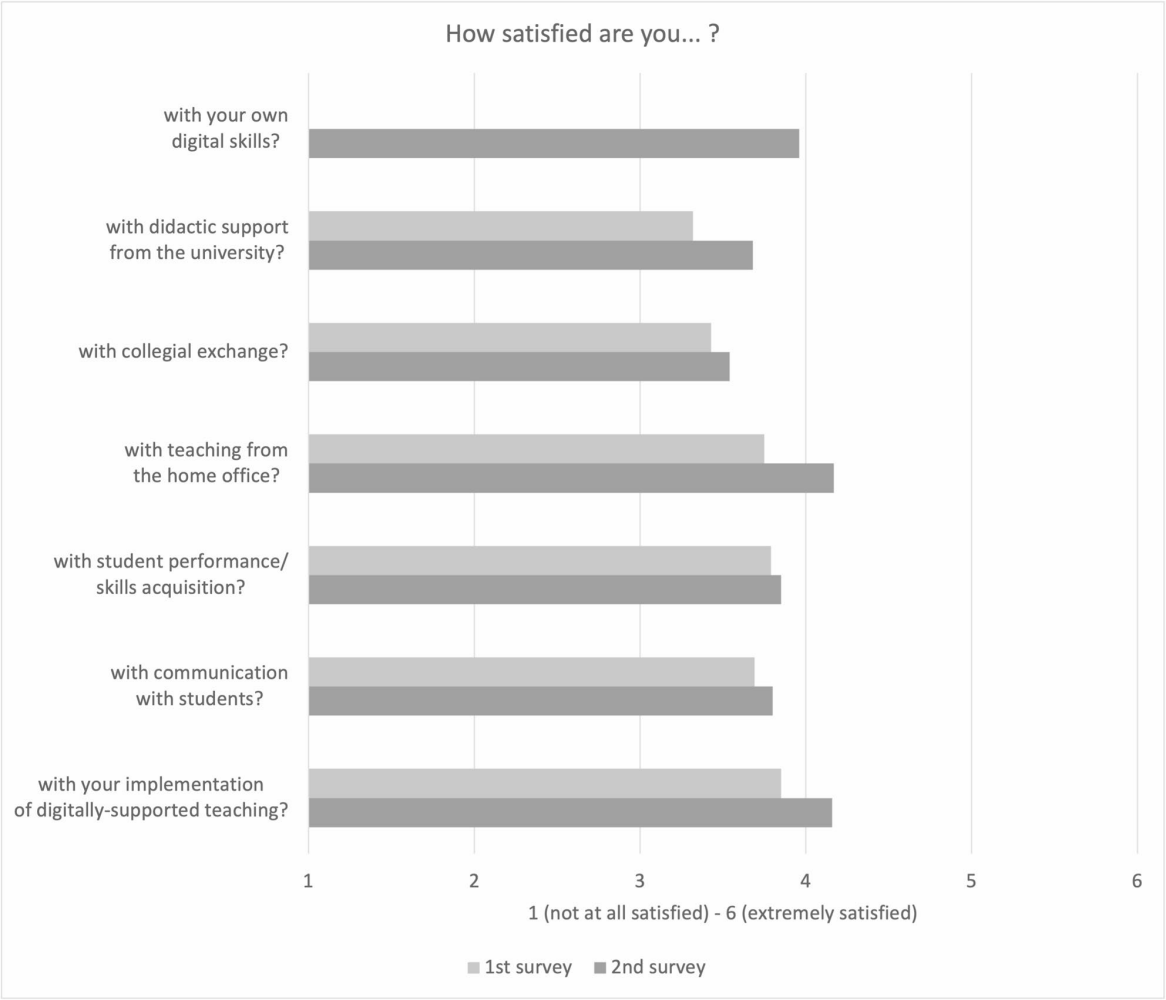
Satisfaction of digital teaching (mean value)

The following results were obtained in relation to the satisfaction of digitally mediated teaching:

- The question regarding satisfaction with respondents’ own digital skills was included only in the second survey; the results indicate that the highest levels of satisfaction were reported at that time (Fig. 4). The descriptive analysis of mean scores across all items reveals positive changes. However, when considering the overall rating scale, these improvements only marginally shift towards a higher evaluative category.
- Satisfaction with teaching from home offices and the implementation of digitally supported teaching methods showed relatively high average scores, suggesting a general adaptation to remote educational environments. In contrast, faculty members reported only moderate satisfaction regarding communication with students, collegial exchange, and didactic support provided by universities, indicating persistent areas for improvement. Ratings for student performance and skills acquisition remained at an average level, reflecting continuous concerns about the effectiveness of digital formats in supporting student learning outcomes.
- The comparative results demonstrate slight improvements from the first to the second survey across most categories, which may reflect increased faculty familiarity and adaptability regarding digital teaching methodologies. Nonetheless, the predominantly moderate satisfaction levels in key areas such as didactic support and student outcomes highlight the continued need for targeted institutional measures and professional development programs.

**Fig. 5:**
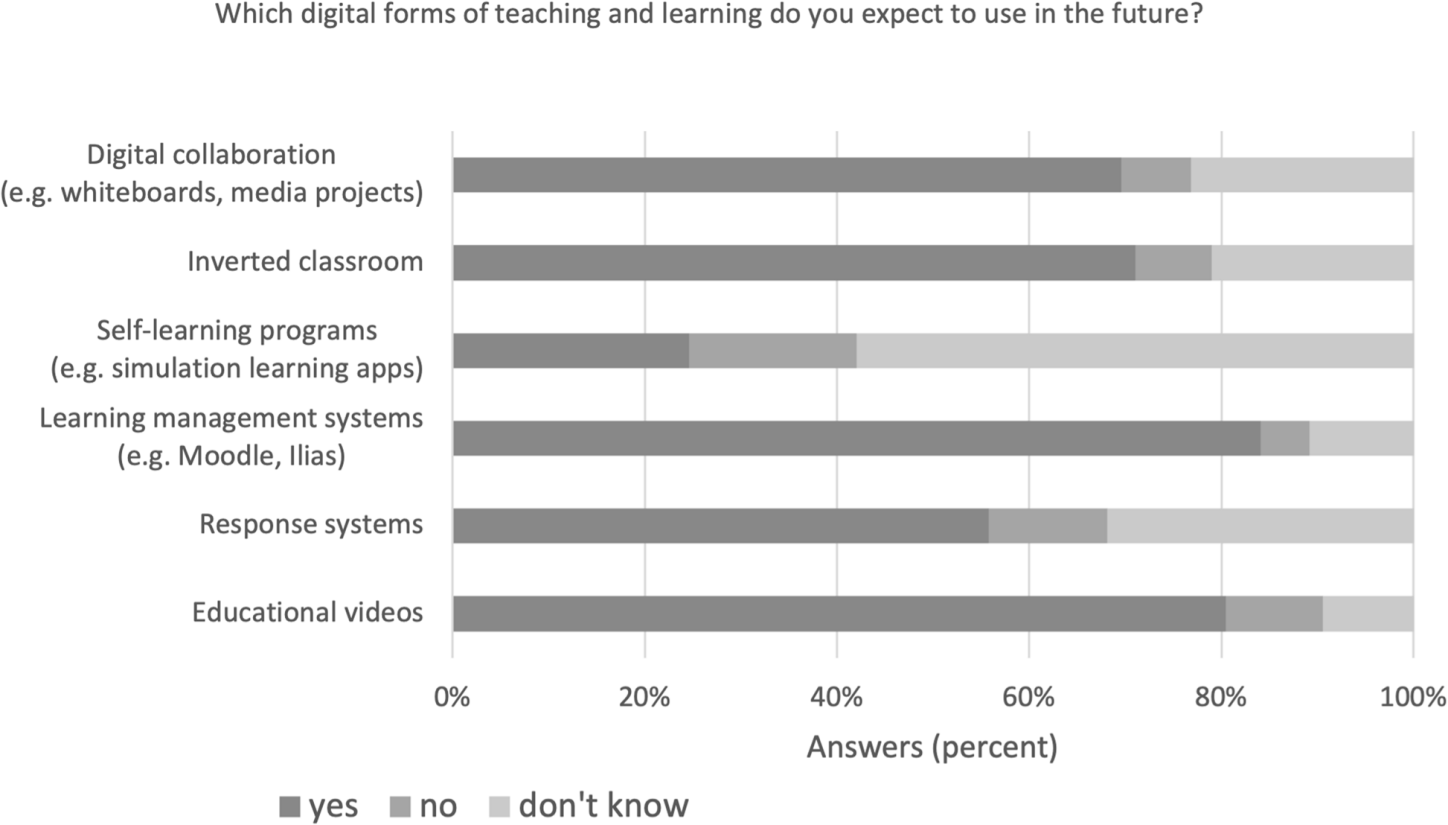
Expectations of future digital teaching and learning forms

The following expectations have been formulated concerning the future utilization of various digital teaching and learning forms and was only collected in the second survey (Fig. 5): Education videos and learning management systems (e.g., Moodle, Ilias) received the highest affirmative responses, indicating strong faculty acceptance and anticipated continued use. Digital collaboration methods (e.g., whiteboards, media projects) and inverted classroom approaches were viewed positively by faculty members, albeit with a degree of uncertainty. The utilization of self-learning programs, incorporating simulation applications, has elicited a range of responses from faculty members, indicative of a prevailing ambivalence or unfamiliarity with such educational methodologies. The response systems demonstrated the highest degree of uncertainty, thus emphasizing the presence of a substantial undecided faculty group, despite the acknowledged potential for enhancing student engagement.

One interesting aspect noted was the willingness to continue digital teaching of health professions at universities—even after the pandemic period: 44 participants said they would use digital teaching in the future, while 30 said they would not.

## Discussion

Study findings reveal that Zoom, Moodle, and MS Teams represent the predominant digital platforms employed in higher education teaching within health sciences/nursing and medical sciences. Their widespread adoption is primarily attributed to institutional and technical mandates rather than deliberate pedagogical choices made by educators. Notably, the continued utilization of digital components—such as educational videos and learning management systems—reflects established acceptance and a perceived effectiveness among university educators. Despite their prevalence, digital tools have largely facilitated content transmission, with comparatively less focus on interactive or collaborative learning strategies. This includes limited adoption of audience response systems, learning apps, and simulation-based applications, which have yet to be leveraged to their full educational potential. The rationale may lie in insufficient didactic innovation—a challenge noted by Tolks et al. [19], Kwon et al. [20], and Marchwacka et al. (2022). Regarding the sustained use of digital teaching methods in the post-pandemic era, results show a heterogeneous response among educators. Slightly more than half of the participants expressed a positive attitude, whereas a considerable fraction exhibited skepticism or reluctance. When reflecting on their overall knowledge and experience with digital learning, educators rated aspects such as enhanced access for physically impaired learners and the attraction of the university more negatively in the second survey than in the first; all other statements received higher approval. Although the advantages of digital teaching are acknowledged, it is perceived as time-intensive, compounded by ambiguous institutional recognition and support structures. These issues highlight a pressing need for reforms in institutional policy, especially concerning the explicit recognition of digital teaching efforts in workload assessments and the establishment of comprehensive technical and didactic support [21–23]. University educators remain doubtful about the efficacy of digital teaching concerning the reduction of drop-out rates and the enhancement of personalized learning experiences [9, 13, 24]. This skepticism underscores the necessity for further research, firmly anchored in empirical methodologies, focusing on the effectiveness of digital teaching approaches and investigating motivational factors and inhibiting barriers to innovative digital tool usage [1, 15, 25]. With the increasing integration of AI technologies in health sciences education, there is an escalating demand for the advancement of faculty digital competencies [12, 26]. Consequently, institutions are prompted to incorporate ethical and legal considerations more intensively into continuing professional development programs, along-side technical skills training.

### Limitations

The study was based on a limited number of cases, which constrains the generalizability of results. Participants were recruited via convenience sampling rather than randomized selection, potentially introducing selection bias. Statistical significance of differences between data collection waves could not be reliably established, as some individuals participated in both surveys while others only attended one. This precluded a clear distinction between paired and unpaired samples, further limiting the robustness of quantitative comparisons.

## Recommendations

To effectively advance digital higher education in healthcare, institutions must prioritize focused faculty development in the use of interactive digital tools and teaching methodologies. In order to facilitate the sustainable and successful integration of digital teaching methods, it is imperative that specific didactic qualifications, digital competence and institutional frameworks are in place [27]. Moreover, the implementation of systematic evaluation strategies is imperative to assess the long-term impacts of digital teaching on student learning outcomes. The exploration and integration of emerging technologies, such as artificial intelligence, has the potential to significantly enhance educational effectiveness in healthcare professions.

## Supplementary Information

Supplementary Material 1.

Supplementary Material 2.

## Data Availability

The original data set for both surveys is provided as an e-supplement to this submission in SPSS and CSV file formats without restriction.
